# The Comparison of Foveal Sensitivity Between Diabetic and Non-diabetic Patients by Using Standard Automated Perimetry 10-2 Protocol: A Cross-Sectional Study

**DOI:** 10.7759/cureus.36981

**Published:** 2023-03-31

**Authors:** Vijaya Sahu, Snehal Kharole

**Affiliations:** 1 Department of Ophthalmology, All India Institute of Medical Sciences, Raipur, IND; 2 Department of Ophthalmology, Shri Bhavani Multispecialty Hospital and Research Institute, Nagpur, IND

**Keywords:** diabetes mellitus, pattern standard deviation, mean deviation, foveal sensitivity, standard automated perimetry

## Abstract

Purpose

The purpose of the study is to assess whether standard automated perimetry (SAP) was capable of detecting early neuroretinal changes by comparing foveal sensitivity in diabetic and non-diabetic subjects.

Settings and design

This is an observational and cross-sectional study that compared foveal sensitivity between a case group of 47 subjects with no or mild-to-moderate diabetic retinopathy (DR) without maculopathy and a control group of 43 healthy subjects.

Materials and Methods

After a thorough ocular examination, all patients were put through tests using a Humphrey visual field analyzer with the Swedish interactive threshold algorithm (SITA) standard system (10-2 software). The primary indicator of success was the age-adjusted foveal awareness-esteem difference. Mean deviation (MD) and pattern standard deviation (PSD) readings were the supplementary performance indicators.

Results

The mean age of the case and control group was 50.76 ± 13.20 years and 49.90 ± 12.20 years, respectively. The probability of cataract development was higher in the case group (p < 0.0001). In the control group, 95.3% had best-corrected visual acuity (BCVA) in the category of good visual acuity (VA) (p < 0.0001). The mean foveal sensitivity in the case group was 28.57 ± 7.54 and 32.16 ± 7.09 for the control group, and the difference was statistically significant (p < 0.023). The mean of MD in the case group was -6.05 ± 7.93, whereas in the control group, it was -3.28 ± 1.70, which was found significant (p = 0.027). There was no difference in PSD between the study groups.

Conclusions

Foveal sensitivity decreased in diabetics, even without maculopathy, so SAP helps identify a patient at risk of future vision loss.

## Introduction

Diabetes mellitus (DM) is one of the world's greatest health challenges, and its prevalence appears to be increasing. In the 1980s, about 108 million population had DM, which had increased to 422 million according to a recent survey by the World Health Organization. Around 8.5% of the world's adult population has DM [1].

By 2000, India now leads in the world with the highest number of DM. Globally, the prevalence of diabetes is predicted to double by 2030, with the maximum increase in India [2-4]. The prevalence of diabetic retinopathy (DR) is also on the rise, with recent studies suggesting that the overall prevalence is as high as 35% for any DR, 7% for proliferative DR, and roughly 7% for diabetic macular edema (DME) [5]. In DR, DME is the most common complication of retinopathy, and it may not be related to how severe the illness is. The diagnosis of DME is complicated by the fact that it is difficult to identify morphological and functional abnormalities in the early stages that are linked to later visual outcomes.

Classically DR, as well as DME, is considered a microvascular complication secondary to hyperglycemia. However, we have now some animal studies, as well as human studies, that demonstrated that neurodegeneration in the retina is equally responsible for the progression of DR and DME, as evidenced by the apoptosis of inner retinal neurons and the shrinking of the ganglion cell layer and nerve fiber layer, and that this early neurodegeneration could be identified prior to the development of obvious vascular sores [6-8]. This neurodegeneration causes decreased foveal sensitivity even when there is no clinically evident DR. Foveal responsiveness (RS) refers to central retinal awareness. Receptive oxygen species and angiotensin II, which affect the transmission of the electrical sign of photoreceptors to the focal sensory system, contribute to the attenuation of RS in DR [9]. So, tests that can detect useful accidents are critical because they should be legible even to those with normal visual acuity (VA) and no history of diabetic maculopathy. Psychophysical examinations, such as tests for visual understanding, are the gold standard for detecting this neuroretinal deficiency, e.g., perimetry, contrast sensitivity, color vision, and electrophysiological tests such as electroretinograms [10].

While visual acuity is often used as a diagnostic tool for visual impairment, it lacks prognostic power in the early stages of the illness since it does not alter fundamentally until around 55% of all neuroretinal channels are damaged [11]. As mentioned earlier, RS decreases in DM, and it correlates with neuroretinal degeneration. These neuroretinal changes can be reflected in a perimetry test. Standard automated perimetry (SAP) is the most commonly done test for this since the 1990s [12]. It gives information about reduced RS, which corresponds well with the area of retinal non-perfusion in DR patients [13]. Many studies demonstrated that the number of points with reduced RS increases with retinopathy severity and was supported by reduced mean deviation (MD) value, decreased foveal sensitivity, and elevated pattern standard deviation (PSD) value. They also showed that DR progression may be predicted using SAP [14-16].

In last few years, microperimetry (MP), also known as fundus-related perimetry, has gained popularity, and it is the established way of macular testing for many diseases. It is another form of automated perimetry. This is used to quantify retinal awareness at a specific retinal location with high test-retest consistency. By taking fundus pictures and measuring retinal sensitivity in the central 20 degrees of the macula at the same time, it provides a strong correlation between anatomical and functional data. Patients with parafoveal or unstable fixation benefit the most from this technique. Several studies have shown that MP is a helpful method for identifying retinal function decline in its earliest stages and thus can identify early macular dysfunctions. The only drawback of MP is the high cost of the device. So, SAP, by using a 10-2 protocol, can be used as a surrogate measure to identify early changes in the function of fovea in diabetic patients [17-19].

The literature search revealed that very few longitudinal studies had been done to establish the effectiveness of these investigations. The current research aimed to prove the value of SAP for the early identification of retinal impairment by comparing foveal sensitivity, MD, and PSD values between diabetic and non-diabetic age-matched healthy individuals. So, it can be used as a cost-effective screening method for predicting the development of DR or for its severity along with blood sugar levels and glycosylated hemoglobin (HbA1c).

## Materials and methods

The study was an observational, cross-sectional, comparative study, conducted between January 2017 and June 2018. Before the commencement, the research was conducted in accordance with the Declaration of Helsinki and was sanctioned by the institutional ethics board of All India Institute of Medical Sciences with the number MC/Ethics/133. Convenient sampling was performed, which included 90 subjects between 18 and 75 years of age. Study subjects were enrolled in two groups. Subjects with diabetes mellitus were assigned to the case group, and those without diabetes mellitus were assigned to the control group. The American Diabetes Association criteria were used for defining diabetes mellitus [20]. The case group included 47 subjects with DM (type 1 and type 2) with and without retinopathy, with no clinically detectable maculopathy, which was further confirmed by optical coherence tomography. These individuals were further classified into those with mild cases of non-proliferative diabetic retinopathy (NPDR) and those with severe cases using the International Clinical Disease Severity Scale for DR [21].

Forty-three patients of a similar age were included as a control group, all of whom were healthy and free of ocular and systemic diseases. Each participant had to give up one of their eyes. In the event that both eyes showed signs of retinopathy, the one with the greater best-corrected visual acuity (BCVA) and milder retinopathy was chosen. If both eyes had the same level of vision, a random eye was chosen. All patients were equally distributed in both groups according to age and sex. The selected subjects were briefed about the nature of the study, and informed consent was obtained. The presence of a mild-to-moderate degree of cataract (lens opacities grading system: nuclear sclerosis (NS) grades II-III and posterior subcapsular cataract (PSC) grade I) was acceptable in both groups [22]. The exclusion criteria for all groups were advanced diabetic retinopathy or treated cases of Diabetic Retinopathy, uncooperative patients, or patients unable to appreciate perimetry stimulus with defective near-vision or any other ocular disease affecting the visual field such as glaucoma. The evaluation used a Humphrey Field Analyzer 750 (Carl Zeiss Meditec, Inc., Dublin, CA) equipped with a Swedish interactive threshold algorithm (SITA) standard 10-2 software with Goldmann III boosts of varying sizes. 10-2 protocol measures 10 degrees temporally and nasally and tests a total of 68 points aligned two degrees apart. The foveal sensitivity, MD, and PSD values printed on the visual field printout with an indication of the probability (e.g., P < 10%, P < 5%, P < 1%, and P < 0.5%) were taken for analysis. Foveal sensitivity is the patient's visual sensitivity (in decibels) on the fovea. MD is a global index that quantifies the patient's visual field as a whole and represents the average deviation from normal age-matched sensitivity values for all of the tests. A negative MD value indicates overall reduced visual field sensitivity. PSD is a global index that quantifies localized visual field sensitivity loss [23].

Procedure

Before starting the examination, all current data were recorded including visual acuity (VA) by using a Snellen chart, refraction, slit-lamp examination, condition of the optical media, and fundus. Data on best-corrected visual acuity (BCVA) were collected and sorted into four groups: excellent VA (6/6 to 6/9), mild visual impairment (6/12 to 6/18), moderate visual impairment (6/24 to 6/60), and severe visual impairment (less than 6/60). The research was conducted in a room that met all of the following criteria: It was large enough to accommodate all of the participants, it was dark enough to prevent any distracting light or noise from seeping in from the outside, it had adequate ventilation to keep the participants cool, and the chairs were adjustable to allow for optimal participant positioning. Both fixation and pupillary diameter were recorded as a part of the procedure. When the percentage of incorrect fixations was fewer than 20%, the test was declared accurate. The examination's results and any abnormalities were meticulously recorded right afterward.

Outcome measure

The primary outcome measure was the difference in age-corrected retinal sensitivity value for fovea (foveal sensitivity). The difference in MD value and PSD values was secondary outcome measures.

Statistical analysis

Microsoft Excel for Windows (2017 version) (Microsoft® Corp., Redmond, WA) was used to input all data, and Statistical Package for Social Sciences (SPSS) (IBM SPSS Statistics, Armonk, NY) was used to run all analyses. Statistics such as the mean, standard deviation, and rate were applied to both continuous and discrete data sets. Student's t-tests or Mann-Whitney U-tests were used to compare the two groups over time. The approach for clusters on all explanatory variables was analyzed using the chi-square test or Fischer's cautious test. In the context of determining the significance of a piece of data, p-values below 0.05 were considered very significant.

## Results

Ninety subjects were recruited during the study period and were divided into two groups. The case group consisted of 47 diabetic patients with no retinopathy or mild-to-moderate NPDR with no clinically evident maculopathy, and the other group was the control group, which included 43 age-matched healthy non-diabetic patients with no systemic illness. The mean age of the case group was 50.76 ± 13.20 years, and that of the control group was 49.90 ± 12.20 years. The majority of the subjects in both groups were from 31 to 50 years of age, i.e., 48.9% (44 out of 90). In the case group, 57.4% were male, and 42.6% were female. In the control group, males were 67.4%, and females were 32.6%. This implies that the two groups were matched for gender and both groups showed male preponderance. In the case group, 51.1% (24 out of 47) subjects had best-corrected visual acuity in the category of good VA, followed by 29.8% (14 out of 47) in the category of mild visual impairment, and 19.2% (nine out of 47) had moderate visual impairment. In the control group, the majority of the subjects had BCVA in the category of good VA, which is 95.3%, and this difference was statistically significant (p < 0.0001) (Table 1).

**Table 1:** Comparison of different variables between study groups BCVA, best-corrected visual acuity; VI, visual impairment; NS, nuclear sclerosis; NPDR, non-proliferative diabetic retinopathy

Variables	Case group	Control group	P value
Number of subjects	47	43	
Gender			
Male	27 (57.4%)	29 (67.4%)	0.329
Female	20 (42.6%)	14 (32.6%)	
BCVA			
Good vision	24 (51.1%)	41 (95.3%)	<0.0001
Mild VI	14 (29.8%)	1 (2.3%)	
Moderate VI	9 (19.2%)	1 (2.3%)	
Cataract grading			
No cataract	4 (8.6%)	19 (44.2%)	<0.0001
NS 1	13 (27.62%)	12 (27.9%)	
NS 2	16 (34.04%)	6 (13.8%)	
NS 3	14 (29.7%)	6 (13.8%)	
Grades of diabetic retinopathy			
No retinopathy	26 (55.3%)	43 (100.0%)	<0.0001
Mild NPDR	3 (6.4%)	0 (0%)	
Moderate NPDR	18 (38.3%)	0 (0%)	

Cataract was present in 91.48% of the subjects in the case group, but it was 55.81% in the control group (Table 1). In the case group, 55.3% of the subjects (26 out of 47) had no retinopathy on fundus examination, followed by moderate NPDR in 38.3% (18 out of 47) and mild NPDR in 6.38% of the subjects (three out of 47), whereas in the control group, 100% were having no fundus changes (Table 1).

The mean foveal sensitivity in the case group was 28.57 ± 7.54 and 32.16 ± 7.09 for the control group. As compared to the normative group, the situational group had significantly less foveal edge awareness on average (Table 2).

**Table 2:** Comparing the mean (±standard deviation) of mean deviation, pattern standard deviation, and foveal sensitivity between study groups

Variables	Groups	No	Mean	Standard deviation	Standard error of the mean	Z	P value
Mean deviation (dB)	Control	43	-3.28	1.70	0.26	-1.28	0.027
	Case	47	-6.05	7.93	1.16		
Pattern standard deviation (dB)	Control	43	1.90	1.55	0.24	-1.68	0.098
	Case	47	2.58	2.25	0.33		
Foveal threshold (dB)	Control	43	32.16	7.09	1.08	-2.32	<0.023
	Case	47	28.57	7.54	1.10		

The mean of MD value in the case group was -6.05 ± 7.93, whereas in the control group, it was -3.28 ± 1.70. There was a statistically significant difference between the case and control groups with respect to the mean of MD value (p = 0.027). The mean PSD in the case group was 2.58 with standard deviation of ±2.25 and 1.90 with standard deviation of ±1.55 for the control group. Statistically, the case group had a greater PSD than the control group, but the difference was not significant (Table 2).

There were no classical visual field defects in the case group, but we found a statistically significant difference in terms of foveal threshold values and mean deviation between both the study subjects. The pattern standard deviation did not show statistically any significance in the present study.

## Discussion

Early macular changes in diabetic patients are difficult to identify clinically by routine ophthalmological checkups. To evaluate early macular changes before being clinically evident, the present study was conducted. The review's case and control groups were not significantly different in terms of age or sexual orientation, but there was clear male dominance in both. There was a significant difference in visual acuity between the two groups, with the situation group doing much worse. The item that mattered was really important, and this result was not supported in the same context by Sampson et al. [24] and Bengtsson et al. [14].

In the present study, out of 47 in the case group, cataract was present in 63.33%, while in the control group, it was 70.21%. We used the Lens Opacities Classification System III to classify cataracts that were contradictory to Lens Opacities Classification System II used by Bengtsson et al. [14]. It was shown that diabetics had a greater risk of developing cataracts than the general population. Our observation was supported by Lutze and Bresnick [25], Li et al. [26], Li et al. [27], and Olafsdottir et al. [28]. There was no classical field defect in the case group of the present study, and the results were similar to the study done by Henricsson and Heijl [29] where they found no field loss in mild retinopathy but evident in advanced stages. Although we did not find any field defects, still, we found some differences in study subjects that were statistically significant, which can be a useful parameter for the early detection of macular changes such as MD and foveal sensitivity value. As for the severity of DR, we used the Global Clinical Disease Reality Scale, whereas Bengtsson et al. [14] classified the severity of DR using the Early Treatment Diabetic Retinopathy Study (ETDRS) system. The current investigation demonstrated a statistically significant difference between the case and control groups in terms of foveal sensitivity levels. The results from our study are consistent with those of Wisznia et al. [30], Sampson et al. [24], Somilleda-Ventura et al. [31], and Joltikov et al. [23].

In the present study, if we compare the case group to the control group, we find that their mean of mean deviation (MD) is much larger and more undesirable. A similar result was seen by Verrotti et al. [32] and Bengtsson et al. [14]. A negative MD value indicates depressed sensitivity, considering that a global list MD value of 0 dB corresponds to a standard field and approximately -30 dB corresponds to a blind field. In the present study, PSD was high in both groups, and a high PSD indicates an irregular hill of vision. In the present study, PSD proved to be of no statistical significance. Similar to the findings of Parravano et al. [33], whereby they discovered statistically significant changes in MD but not in PSD, we too discovered such a difference in our own investigation. Nevertheless, no significant change in PSD was seen. Trick et al. [12] proved contradictory to our study stating that mean deviation showed no statistical difference but there was a statistically significant difference in pattern standard deviation.

Some of the limitations of the study include a small sample size and a nonrandomized study; long follow-ups will be better, so a cohort study would be better.

## Conclusions

Our study concluded that foveal sensitivity was significantly low in diabetic patients, even without clinically evident macular involvement. So, SAP can detect functional changes in the fovea before any significant structural changes and can be used as a screening method for identifying individuals who are at risk of losing vision in the future, thus helping in reducing visual morbidity.
